# Alpha- and Gamma-Tocopherol Modulates the Amyloidogenic Pathway of Amyloid Precursor Protein in an *in vitro* Model of Alzheimer’s Disease: A Transcriptional Study

**DOI:** 10.3389/fncel.2022.846459

**Published:** 2022-05-05

**Authors:** Aslina Pahrudin Arrozi, Siti Nur Syazwani Shukri, Nuraqila Mohd Murshid, Ahmad Baihaqi Ahmad Shahzalli, Wan Zurinah Wan Ngah, Hanafi Ahmad Damanhuri, Suzana Makpol

**Affiliations:** Department of Biochemistry, Faculty of Medicine, Universiti Kebangsaan Malaysia Medical Centre, Cheras, Malaysia

**Keywords:** tocopherol isomers, amyloid beta, APP–amyloid precursor protein, gene expression, Alzheimer’s disease

## Abstract

The amyloid precursor protein (APP) processing pathway was altered in Alzheimer’s disease (AD) and contributed to abnormal amyloid-beta (Aβ) production, which forms insoluble interneuron protein aggregates known as amyloid plaques in the brain. Targeting the APP processing pathway is still fundamental for AD modifying therapy. Extensive research has evaluated the protective effects of vitamin E as an antioxidant and as a signaling molecule. The present study aimed to investigate the modulatory effects of different tocopherol isomers on the expression of genes involved in regulating the APP processing pathway *in vitro*. The screening for the effective tocopherol isomers in reducing APP expression and Aβ-42 was carried out in SH-SY5Y stably overexpressed APP Swedish. Subsequently, quantitative one-step real-time PCR was performed to determine the modulatory effects of selected tocopherol isomers on the expression of genes in SH-SY5Y stably overexpressed three different types of APP (wild-type, APP Swedish, and APP Swedish/Indiana). Our results showed that all tocopherol isomers, especially at higher concentrations (80–100 μM), significantly increased (*p* < 0.05) the cell viability in all cells group, but only α-tocopherol (ATF) and γ-tocopherol (GTF) significantly decreased (*p* < 0.05) the APP mRNA level without statistically significant APP protein level, accompanied with a reduced significance (*p* < 0.05) on the level of Aβ-42 in SH-SY5Y APP Swedish. On the other hand, β- and δ-tocopherol (BTF and DTF) showed no effects on the level of APP expression and Aβ-42. Subsequent results demonstrated that ATF and GTF significantly decreased (*p* < 0.05) the expression of gene beta-site APP cleaving enzyme (*BACE1*), *APH1B*, and Nicastrin (*NCSTN*), but significantly increased (*p* < 0.05) the expression of Sirtuin 1 (*SIRT1*) in SH-SY5Y stably expressed the mutant APP form. These findings suggested that ATF and GTF could modulate altered pathways and may help ameliorate the burden of amyloid load in AD.

## Introduction

The primary pathological hallmarks of Alzheimer’s disease (AD) are extracellular amyloid plaques and intracellular neurofibrillary tangles (NFTs) in the brain tissues of patients with AD (DeTure and Dickson, 2019). In the central nervous system (CNS), the enzymatic cleavage of amyloid precursor protein (APP) by α-secretase within the Aβ domain precludes the Aβ generation and releases of a sizeable soluble ectodomain of APP called sAPPα. Whereas, the enzymatic cleavage of APP protein by β-secretase followed by γ-secretase promoted the generation of Aβ peptide with the majority of species as Aβ-42, which has a high tendency to form aggregates and is thus considered the most toxic form (Zhang et al., 2011).

The α-secretase is a zinc metalloproteinase encoded by the gene of several members of the *a disintegrin and metalloproteinase (ADAM)* family, such as *ADAM9*, *ADAM10*, and *ADAM17* (Allinson et al., 2003). Based on the significant abolition of sAPPα generation in mice with neural ADAM10 conditionally knocked out (Jorissen et al., 2010) and strong correlation between reduced ADAM10 and decreased sAPPα in the platelets and cerebrospinal fluid of sporadic AD patients (Colciaghi et al., 2002), ADAM10 was strongly suggested as the constitutive α-secretase that is active at the cell surface. Recent evidence indicates that Sirtuin 1 (SIRT1), along with its varied role in the regulation of aging, is also a key regulator of α-secretase and, thus, the non-amyloidogenic processing (Guo et al., 2016; Manjula et al., 2021). The major β-secretase encoded by the gene beta-site APP cleaving enzyme (*BACE1*) and the catalytic activity of γ-secretase complex that was encoded by at least four genes, namely, presenilin (*PS*, *PS1*, or *PS2*), Nicastrin (*NCSTN*), anterior pharynx-defective-1 (*APH1*), and presenilin enhancer-2 (*PEN-2*) (De Strooper, 2003; Kimberly et al., 2003), was the key regulator in the amyloidogenic processing (Cole and Vassar, 2007). Patients suffering from AD have shown an approximately 30% increase in BACE1 levels and activity vs. normal subjects (Holsinger et al., 2002; Yang et al., 2003). A previous study also reported that dysregulated expression levels of APH1B in peripheral blood were associated with brain atrophy and amyloid-β deposition in AD (Park et al., 2021). In contrast, the inactivation of NCSTN restricts amyloid deposition in an AD mouse model (Sesele et al., 2013), suggesting these genes’ critical role in Aβ production. As the APP processing is altered in AD (O’Brien and Wong, 2011), targeting this pathway is still as fundamental as AD modifying therapy (Zhao et al., 2020).

Vitamin E was proposed as a treatment for AD many years ago. However, the effectiveness is still not apparent. It acts as an antioxidant, anti-inflammatory, and has hypocholesterolemic properties and signaling molecules, driving its importance for brain health (Lloret et al., 2019). Natural vitamin E exists in two major groups, tocotrienol and tocopherol, both consisting of four isomers (i.e., α, β, γ, δ). α-tocopherol (ATF) is the most widely studied as it has the highest biological activity in the human body (Drevon, 1991). However, γ-tocopherol (GTF) was demonstrated to have unique functions unrelated to ATF, such as reducing the reactive nitrogen species in the brain (Williamson et al., 2002). A previous study in our laboratory demonstrated that treatment with ATF and GTF reduced the level of reactive oxygen species (ROS) in the mitochondria, preventing apoptosis and improving mitochondrial functions such as increased respiratory capacity and membrane potential, eventually expand the production of energy in the form of ATP (Pahrudin Arrozi et al., 2020, 2021). However, their mechanism of action as an anti-amyloid is not yet fully elucidated. Therefore, the present study investigated the role of tocopherol isomers on APP gene and protein, APP-processing-related genes, and Aβ-42 production.

## Materials and Methods

### Development of SH-SY5Y Cells Stably Expressing the Amyloid Precursor Protein Wild-Type, Amyloid Precursor Protein Swedish and Amyloid Precursor Protein Swedish/Indiana

The development of SH-SY5Y cells stably expressing the APP wild-type (APP WT), APP Swedish (APP Swe), and APP Swedish/Indiana (APP Swe/Ind) has been described in our previous study (Pahrudin Arrozi et al., 2017). Briefly, SH-SY5Y cells were transfected with three different plasmids carrying the wild-type (WT), swedish (Swe), or swedish/Indiana (Swe/Ind) form of *APP* gene using Lipofectamine 3,000 (Life Technologies, Carlsbad, CA, United States), following the protocol from the manufacturer. After 72 h of transfection, the positive green fluorescent protein (GFP)-expressing cells were selected using a selection medium containing 400 μg/ml of geneticin (G418) (Life Technologies, United States). The level of Aβ-40 and 42 in each cell group was also examined, which showed that the ratio of Aβ42/40 was increased in the following order: SH-SY5Y APP WT < SH-SY5Y APP Swe < SH-SY5Y APP Swe/Ind (Pahrudin Arrozi et al., 2017).

### Cell Culture

The non-transfected SH-SY5Y cells were cultured in a complete culture medium (CCM) containing a 1:1 ratio of Dulbecco’s modified Eagle’s medium (DMEM) and Ham’s F-12 medium (Gibco, Life Technologies, United States) supplemented with 1% penicillin/streptomycin (Gibco, Life Technologies, United States) and 10% fetal bovine serum (FBS) (HyClone, Logan, UT, United States). The stably transfected SH-SY5Y cells were cultured in CCM without penicillin/streptomycin and with 400 μg/ml of geneticin (Gibco, Life Technologies, United States). All cell types were cultured in a humidified atmosphere of 5% CO2 at 37°C.

### Treatment With Tocopherol Isomers

The tocopherol isomers were obtained from ChromaDex, Los Angeles, CA, United States. The stock solution of 0.5 M for each isomer was freshly prepared with 100% undenatured ethanol and stored at −20°C for approximately 1 month. The undenatured ethanol was used as it has no additives and denaturants. Before treatment, 15 μl of tocopherol isomer from the 0.5 M stock was pre-incubated with 20 μl FBS overnight. The incubation with serum is necessary as the protein constituents must deliver this water-insoluble compound to cells (Sontag and Parker, 2007). Then, 18 μl of CCM and 21 μl of 100% undenatured ethanol were added to the activated isomer to make a 0.1 M stock solution. Next, further dilution was carried out to 0.05 M with 72 μl of a mixture of CCM and undenatured ethanol in a 1:1 ratio. After that, a stock solution of 0.025 M was prepared with 146 μl of CCM. Next, 60 μl from 0.025 M stock was taken out and added with 240 μl of CCM to make a 0.05 M stock solution. Finally, the desired working concentration of tocopherol isomer was prepared for 1, 3, 5, 10, 20, 40, 60, 80, and 100 μM. The final concentration of undenatured ethanol and FBS was kept constant (below 0.1%) (calculated from the overnight incubation with 20 μl of FBS). The treatment was carried out for 24 h.

### Cell Viability Assay

The concentration of β- (BTF) and δ- tocopherol (DTF) from 1 to 100 μM were screened by cell viability assay using CellTiter 96 Aqueous Non-Radioactive Cell Proliferation Assay (Promega, Madison, WI, United States). The kit supplemented 3-(4,5-dimethylthiazol-2-yl)-5-(3-carboxymethoxyphenyl)2-(4-sulphonyl)-2H-tetrazolium (MTS) and the electron coupling agent phenazine methosulfate (PMS). The dehydrogenase enzyme reduced the MTS compound into a formazan product soluble in the medium in active cells. This colored formazan product is proportional to the number of viable cells. Briefly, the MTS solution was premixed with media at the ratio of 2:3 before adding 50 μl of the solution to each well of the cells and incubated in a humidified incubator at 37°C in 5% CO_2_ for 2–4 h. The quantity of formazan product was determined by measuring the absorbance at 490 nm with a microplate reader (Enspire, Perkin Elmer, Waltham, MA, United States).

### Quantitative Real-Time PCR

According to the manufacturer’s protocol, RNA extraction was carried out with TRI Reagent^®^ (Molecular Research Centre, Cincinnati, OH, United States). qRT-PCR was performed using the KAPA SYBR FAST One-Step qPCR kit (Kapa Biosystems, Boston, MA, United States). Each sample was tested in triplicate. The expression level of each gene was normalized to that of GAPDH. The relative transcription was analyzed by the 2^–ΔΔCT^ method. The fold change was calculated relative to control non-transfected when comparing the group of cells before treatment. The fold change was calculated relative to untreated control when comparing different isomers in each group of cells. The primers used in this study are listed in Table 1.

**TABLE 1:** The list of primers.

Gene	Primer sequences
*APP*	5′-GCTGGTGGAGACACACATGGCC-3′ (forward) 5′-GGATCTGAGCGGCTTTCTTGGG-3′ (reverse)
*ADAM10*	5′-CGATGTGCTGCTGTTAACCC-3′ (forward) 5′-TCCCATACTGACCTCCCATCC-3′ (reverse)
*BACE1*	5′-CGCTCCTAATGGTACGTGGG-3′ (forward) 5′-AGGAGTAGGATGCAGTGGGT-3′ (reverse)
*NCSTN*	5′-ATCTCTGGGCTGAGCCTACT-3′ (forward) 5′-TACAACTGAGCACTGTGGGC-3′ (reverse)
*APH1B*	5′- ACCTGGGCATTCTTAGCTGC-3′ (forward) 5′- GGTTATCTGGAGCGCTGGTT-3′ (reverse)
*SIRT1*	5′-GGAGCAGATTAGTAGGCGGC-3′ (forward) 5′-GACTCTGGCATGTCCCACTA-3′ (reverse)
*GAPDH*	5′-TCCCTGAGCTGAACGGAAG-3′ (forward) 5′-GGAGGAGTGGGTGTCGCTGT-3′ (reverse)

### Protein Extraction and Western Blot Analysis

Whole-cell extracts were prepared from SH-SY5Y cell cultures. After 24 h treatment with tocopherol isomers, cells were washed in ice-cold phosphate buffered saline (PBS) and removed from the flask’s surface by scraping the cells in radioimmunoprecipitation assay (RIPA) buffer (Thermo Fischer Scientific, Waltham, MA, United States). Cell extracts were sonicated, collected, and stored at −80°C until protein concentration was measured by Bradford assay (Bradford, 1976). According to the manufacturer’s instructions, electrophoresis and blotting were performed using the Mini-PROTEAN^®^ Tetra Cell system (Bio-rad, Hercules, CA, United States). Denatured samples with an equal amount of protein per lane (20 μg) were separated on gradient gels (10% SDS-PAGE gel) electrotransferred on nitrocellulose membrane (GE Healthcare, Marlborough, MA, United States). They blocked in 5% skimmed milk for 1 h at room temperature. The membranes were then incubated overnight with primary antibodies at 4°C (rabbit anti-APP, 1:1,000, Abcam, Cambridge, United Kingdom; mouse anti-β-actin, 1:5,000 (Santa Cruz, Dallas, TX, United States). Before incubation with primary antibody, the membranes were washed in triphosphate buffered saline (TPBS) several times, and then incubated with secondary antibody (anti-rabbit IgG, 1:2,000, Abcam, United Kingdom; anti-mouse, 1:2,000, Abcam, United Kingdom)) for 1 h at room temperature. Peroxidase enzyme activity was detected using WesternBright*™* Sirius Western blotting detection kit (Advansta, Menlo Park, CA, United States) and visualized on Gel-Doc FluorChem FC2 (Alpha Innotech, San Leandro, CA, United States).

### Aβ42 Sandwich ELISA Assay

Conditioned media was collected from cells. Protein inhibitors were added to the media to prevent the degradation of Aβ protein. According to the manufacturer’s instructions, the concentration of Aβ42/40 was determined by using human Aβ42 or Aβ40 ELISA kit (Elabscience, Wuhan, China).

### Statistical Analysis

Data obtained were expressed as mean ± SD, and statistical analysis was carried out using GraphPad PRISM v8 software (GraphPad Software, La Jolla, CA, United States). Statistical comparisons between groups were performed using one-way ANOVA followed by the *post-hoc* test. The statistical significance of all tests was set at *p* < 0.05.

## Results

### Treatment With Tocopherol Isomers at Higher Concentration Increased the Cell Viability in SH-SY5Y Overexpressed Wild-Type and Mutant Amyloid Precursor Protein

**FIGURE 1:**
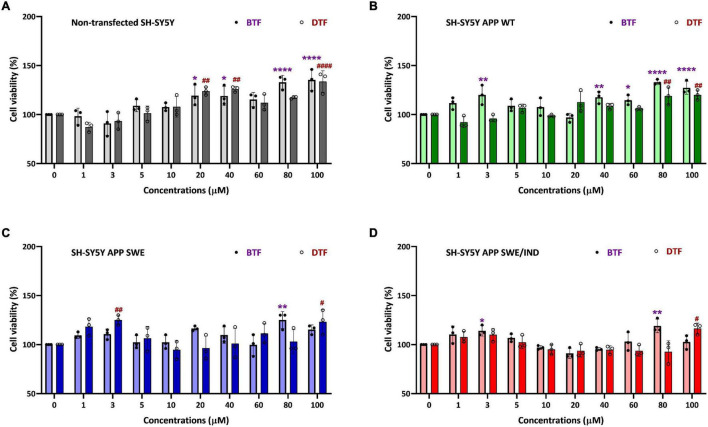
Cell viability assay of tocopherol isomer [β- tocopherol (BTF) and δ-tocopherol (DTF)] treatment from 1 to 100 μM for 24 h in **(A)** Non-transfected SH-SY5Y, **(B)** SH-SY5Y amyloid precursor protein (APP) wild-type (WT), **(C)** SH-SY5Y APP Swedish (Swe), and **(D)** SH-SY5Y APP Swedish/Indiana (Swe/Ind). Data presented as mean ± SD, (*n* = 3). *p < 0.05, **p < 0.01, and ****p < 0.0001 significantly different compared to untreated control in BTF treatment group. ^#^*p* < 0.05, ^##^*p* < 0.01, and ^####^*p* < 0.0001 significantly different compared to untreated control in DTF treatment group.

To determine the cytotoxicity effect of the different tocopherol isomers, a range of doses from 1 to 100 μM were screened by cell viability assay in non-transfected and stably transfected SH-SY5Y with three different types of *APP* gene. In our previous data, the incubation with ATF and GTF for 24 h did not show any significant cytotoxic effect in all groups of cells. In addition, ATF at 5 and 100 μM and GTF at 5 and 80 μM increased the cell viability in SH-SY5Y stably transfected with APP WT, APP Swe, and APP Swe/Ind (Pahrudin Arrozi et al., 2020). The present study also showed similar findings. There was no cytotoxicity effect with BTF and DTF treatment from 1 to 100 μM in all cells. Furthermore, BTF treatment at a lower dose (3 μM) significantly increased the cell viability in APP WT and APP Swe/Ind-expressing cells (*p* < 0.01 and *p* < 0.05 respectively) (Figures 1B,D). Still, DTF treatment at the same dose significantly increased the cell viability only in APP Swe-expressing cells compared to untreated control (*p* < 0.01) (Figure 1C). Moreover, the treatment with BTF at 80 μM and DTF at 100 μM demonstrated a significant increase in cell viability in non-transfected (Figure 1A), APP WT (Figure 1B), APP Swe (Figure 1C), and APP Swe/Ind-expressing cells (Figure 1D) compared to untreated control (*p* < 0.0001, *p* < 0.01, and *p* < 0.05 respectively) in respective groups of cells. Based on these findings, ATF at 5 and 100 μM, BTF at 3 and 80 μM, DTF at 3 and 100 μM, and GTF at 5 and 80 μM were chosen for further screening for their effects on APP expression at gene and protein levels, and also on the level of Aβ-42.

### α-Tocopherol and γ-Tocopherol Efficiently Altered the Amyloid Precursor Protein Expression at the Gene but Not Protein Level Accompanied by Decreased Aβ-42

Previously, we showed that APP gene and protein level increased significantly in SH-SY5Y stably transfected with APP WT or mutant (Swe or Swe/Ind) compared to non-transfected control (Pahrudin Arrozi et al., 2017). On the other hand, the ratio of Aβ42/Aβ40 was shown in the following order: non-transfected SH-SY5Y < SH-SY5Y APP WT < SH-SY5Y APP Swe < SH-SY5Y APP Swe/Ind (Pahrudin Arrozi et al., 2017). As the ratio of Aβ42/Aβ40 in SH-SY5Y expressing APP Swe represent the intermediate level between cells expressing APP WT and APP Swe/Ind, further screening was done to investigate the effects of selected doses of different tocopherol isomers on the level of APP expression and Aβ-42 only in APP Swe-expressing cells. The non-transfected SH-SY5Y act as a control group without APP overexpression.

**FIGURE 2:**
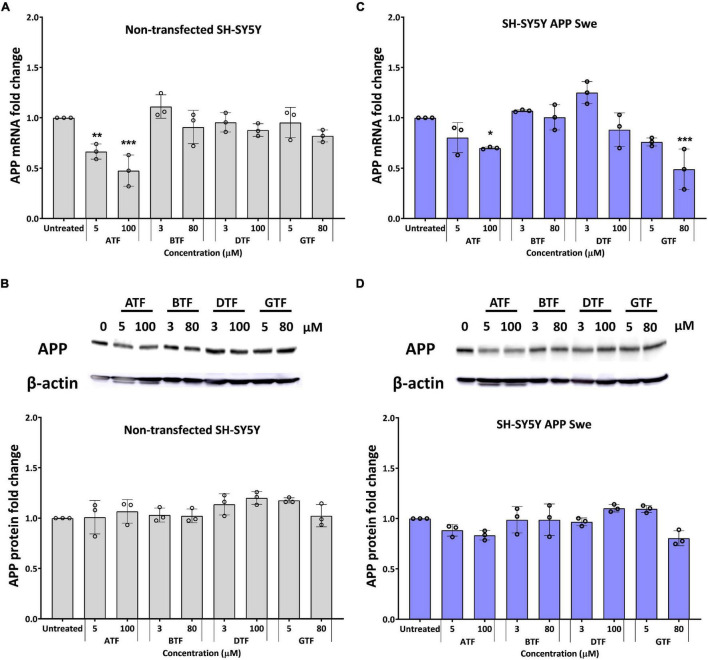
Effect of tocopherol isomer [α-tocopherol (ATF), BTF, DTF, and γ-tocopherol (GTF)] treatment at two concentrations for 24 h on the APP gene expression **(A,C)** and APP protein expression **(B,D)** in non-transfected SH-SY5Y **(A,B)** and SH-SY5Y APP Swe **(C,D)**. Data presented as mean ± SD, (*n* = 3). *p < 0.05, **p < 0.01, and ***p < 0.001 significantly different compared to untreated control in respective group.

The treatment with ATF at 5 and 100 μM significantly decreased the level of the APP mRNA expression (*p* < 0.01 and *p* < 0.001) (Figure 2A), but there were no changes in the level of the APP protein expression (Figure 2B) in non-transfected cells compared to untreated control. Similarly, the treatment with ATF at 100 μM and GTF at 80 μM significantly decreased the level of the APP mRNA expression (*p* < 0.05 and *p* < 0.001, respectively). In contrast, at 5 μM, both treatments only showed a decreasing trend without statistical significance compared to untreated control in APP Swe-expressing cells (Figure 2C). Although reduced significantly at the gene level, treatment with ATF or GTF did not show statistical significance and only showed a slight reduction on the level of the APP protein expression compared to untreated control (Figure 2D). Whereas the treatment with BTF and DTF at selected doses showed no significant changes in the APP gene and protein expression level compared to untreated control in both groups of cells (Figure 2).

**FIGURE 3:**
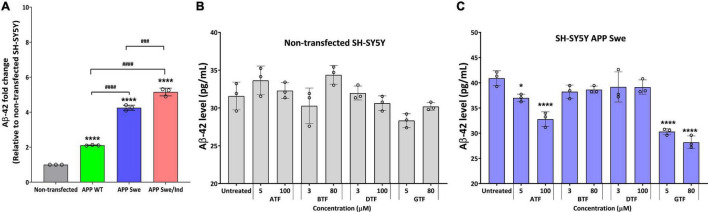
**(A)** The baseline level of Aβ42 in SH-SY5Y is stably transfected with APP WT, APP Swe, and APP Swe/Ind. Effect of tocopherol isomer (ATF, BTF, DTF, and GTF) treatment at two concentrations for 24 h on the level of Aβ42 in **(B)** non-transfected SH-SY5Y and **(C)** SH-SY5Y APP Swe. Data presented as mean ± SD, (*n* = 3). ^###^*p* < 0.001 and ^####^*p* < 0.0001, *p < 0.05 and ****p < 0.0001 significantly different compared to untreated control in respective group.

We further evaluate the effect of the tocopherol isomers on the level of Aβ-42. Before treatment, all types of APP-expressing cells showed a significantly higher level of Aβ42 compared to non-transfected and transfected cells delivered the following order: SH-SY5Y APP WT < SH-SY5Y APP Swe < SH-SY5Y APP Swe/Ind (Figure 3A). There was no significant difference with all tocopherol isomer treatment on the level of Aβ-42 compared to untreated control in non-transfected cells (Figure 3B). Interestingly, the treatment with ATF (5 and 100 μM; *p* < 0.05 and *p* < 0.0001, respectively) and GTF (5 and 80 μM; *p* < 0.0001) significantly decreased the level of Aβ-42, whereas BTF and DTF showed no significant difference compared to untreated control in APP Swe-expressing cells (Figure 3C). Based on these findings, ATF and GTF reduced the level of Aβ42 without affecting APP protein expression. Therefore, investigating their role in the APP processing pathway at the transcriptional level was conducted to elucidate their mechanism of action in SH-SY5Y, further stably expressing the APP WT, single-mutant (Swe), and double-mutant (Swe/Ind).

### α-Tocopherol and γ-Tocopherol Treatment Suppressed the Expression of Genes Involved in the Amyloid Precursor Protein Amyloidogenic Processing Pathway

The expression of several genes involved in the regulation of the APP processing pathway was measured by qPCR. The genes are *ADAM10*, which encodes the major α-secretase in the brain; *BACE1*, which encodes a transmembrane aspartyl protease responsible for β-secretase processing; *APH1B*, which encodes a multi-pass transmembrane protein that is a functional component of the gamma-secretase complex; *NCSTN*, which encodes a type I transmembrane glycoprotein that is an integral component of the multimeric γ-secretase complex; and *SIRT1*, which encodes sirtuin 1, NAD^+^ dependent histone deacetylase.

**FIGURE 4:**
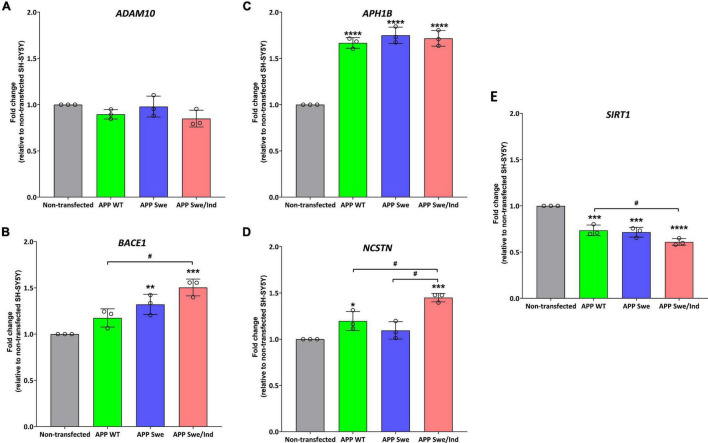
The expression of genes involved in the regulation of the APP processing pathway in SH-SHY stably expressed APP WT, APP Swe, and APP Swe/Ind prior treatment **(A)** *ADAM10*, **(B)** beta-site APP cleaving enzyme (*BACE1*), **(C)** *APH1B*, **(D)** Nicastrin (*NCSTN*) **(E)** Sirtuin 1 (*SIRT1*). Data presented as mean ± SD, (*n* = 3). *p* < 0.05, *p < 0.05, **p < 0.01, ***p < 0.001, and ****p < 0.0001 significantly different compared to non-transfected SH-SY5Y.

Before treatment, the baseline level in each cells group was measured. There was no significant difference between the groups in the expression level of *ADAM10* (Figure 4A). Whereas the expression level of *BACE1* was significantly upregulated in APP Swe and APP Swe/Ind-expressing cells compared to non-transfected cells (*p* < 0.01 and *p* < 0.001, respectively; Figure 4B). The expression level of *BACE1* in APP Swe/Ind was also significantly upregulated compared to APP WT (*p* < 0.05; Figure 4B). Meanwhile, the expression level of *APH1B* was significantly upregulated in all APP type expressing cells compared to non-transfected cells (*p* < 0.0001 for all groups; Figure 4C). Furthermore, the expression level of *NCSTN* was significantly upregulated in APP WT- and APP Swe/Ind-expressing cells compared to non-transfected cells (*p* < 0.05 and *p* < 0.001, respectively). In addition, the level of upregulation in APP Swe/Ind was significant when compared to APP WT and APP Swe-expressing cells (*p* < 0.05 and *p* < 0.01, respectively) (Figure 4D). In contrast, the expression level of *SIRT1* was significantly downregulated in all APP types expressing cells compared to non-transfected cells (*p* < 0.001 for APP WT and Swe, *p* < 0.0001 for APP Swe/Ind), which is the level of downregulation in APP Swe/Ind that was significant when compared to APP WT-expressing cells (*p* < 0.05; Figure 4E). These results suggested that the genes regulating the APP processing pathway were altered in SH-SY5Y cells with overexpressed. A more substantial alteration effect was seen in cells with overexpressed mutant APP.

**FIGURE 5:**
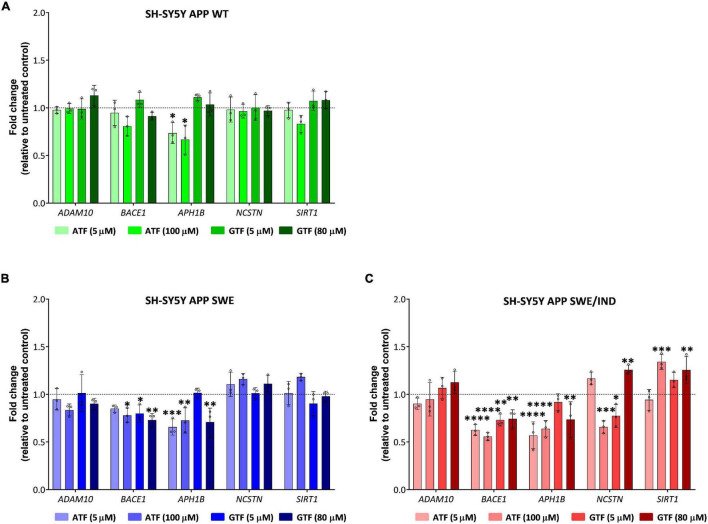
Effect of tocopherol isomer (ATF and GTF) treatment at two concentrations for 24 h on the expression of genes involved in the regulation of the APP processing pathway in **(A)** SH-SY5Y APP WT, **(B)** SH-SY5Y APP Swe, and **(C)** SH-SY5Y APP Swe/Ind. Data presented as mean ± SD, (*n* = 3). *p < 0.05, ^**^p < 0.01, ^***^p < 0.001, and ^****^p < 0.0001 significantly different compared to untreated control in respective cells.

In APP WT-expressing cells, the treatment with ATF at 5 and 100 μM significantly downregulated the expression of *APH1B* without affecting the expression of other genes compared to untreated control (*p* < 0.05; Figure 5A). On the other hand, treatment with GTF did not alter the expression of all candidate genes in the same group of cells (Figure 5A). Meanwhile, in APP Swe-expressing cells, the treatment with ATF and GTF, especially at a higher dose, significantly downregulated the expression of *BACE1* (*p* < 0.05 and *p* < 0.01, respectively) and *APH1B* (*p* < 0.01 for both) compared to untreated control (Figure 5B). The expression of the other genes, such as *ADAM10*, *NCSTN*, and *SIRT1*, was unchanged with ATF or GTF treatments (Figure 5B). However, in APP Swe/Ind-expressing cells, ATF and GTF affected several genes. For example, ATF and GTF significantly downregulated the expression of *BACE1* regardless of the concentration compared to untreated control (*p* < 0.0001 and *p* < 0.01, respectively; Figure 5C). Similarly, the treatment with ATF at both lower and higher doses and GTF at a higher concentration significantly downregulated the expression of *APH1B* compared to untreated control (*p* < 0.0001 and *p* < 0.01, respectively; Figure 5C). Furthermore, the treatment with ATF at a higher concentration also significantly downregulated (*p* < 0.001) the expression of *NCSTN*. Meanwhile, the treatment with GTF showed different effects at different concentrations, such as significantly downregulated (*p* < 0.05) and upregulated (*p* < 0.01) with lower and higher concentration, respectively, compared to untreated control (Figure 5C). In addition, the treatment with ATF and GTF at a higher concentration significantly upregulated the expression of SIRT1 compared to untreated control (*p* < 0.001 and *p* < 0.01, respectively; Figure 5C). There was no significant difference in the expression of ADAM10 with ATF and GTF treatment in all APP types expressing cells compared to untreated control (Figures 5A–C).

According to these results, the treatment with ATF and GTF for 24 h differently modulated the expression of genes involved in regulating the APP processing pathway in SH-SY5Y with different types of overexpressed APP. Specifically, ATF and GTF downregulated the expression of genes involved in mediating the APP processing toward the amyloidogenic pathway, such as *BACE1*, *APH1B* and *NCSTN*, and upregulated the expression of a gene involved in mediating the amyloidogenic pathway, such as *SIRT1*.

## Discussion

In the present study, we demonstrated the treatment with different tocopherol isomers (ATF, BTF, DTF, and GTF) at higher concentrations (80 or 100 μM) and promoted the cell viability in SH-SY5Y with different types of overexpressed APP. However, only ATF and GTF possessed the anti-amyloid property as seen with a reduced level of Aβ42 in SH-SY5Y APP Swe. We also showed that ATF and GTF downregulated the APP expression at the gene level, but not at the protein level. Further investigation found that ATF and GTF downregulated the expression of genes involved in mediating the APP processing toward the amyloidogenic pathway, such as BACE1, APH1B, and NCSTN, and upregulated the expression of *SIRT1* gene, which mediates the non-amyloidogenic pathway.

Before treatment, the cells overexpressed with WT and mutant APP showed upregulation in genes involved in the APP amyloidogenic pathway, such as *BACE1*, *APH1B*, and *NCSTN*, with downregulation of *SIRT1*, which mediates the non amyloidogenic pathway. The degree of up and downregulation was more pronounced in overexpressed mutant cells (Swe or Swe/Ind) than in WT APP. Accordingly, the level of Aβ42 was also more apparent in these mutant APP overexpressed cells than in the WT. The APP Swe corresponds to genetic mutation, which involves a substitution of two amino acids, from lysine (K) and methionine (M) to asparagine (N) and leucine (L), at a point immediately adjacent to the β-secretase site in APP in rare familial AD cases with six times increased Aβ production (Citron et al., 1992; Haass et al., 1995). The major β-secretase was encoded by *BACE1* (Cole and Vassar, 2007). An *in vivo* study using BACE1 (−/−) knockout transgenic mice expressing human APP Swe showed an almost 100% reduction in BACE1 activity. In contrast, the metabolites of the β-secretory pathway, such as sAPPβ, C99, Aβ40, and Aβ42, were not detectable (Rabe et al., 2011). Genetic ablation of BACE1 wholly prevents the amyloid pathology in a transgenic mouse model expressing human APP Swe/Ind mutations (Durrant et al., 2020), indicating the prominent role of the BACE1 gene as underlying mechanisms for Aβ production in mutant APP such as Swe and Swe/Ind.

Meanwhile, the APP Indiana mutation corresponds to the substitution of a single amino acid valine (V) to phenylalanine (F) near the γ-secretase cleavage site (Murrell et al., 1991; Tamaoka et al., 1994). Mechanistically, most of the pathogenic APP mutation near the γ-secretase cleavage site elevates the Aβ42/Aβ40 ratio (Weggen and Beher, 2012), with the most robust data being obtained for the V717 FAD mutants (Suzuki et al., 1994; Bergman et al., 2003; Hecimovic et al., 2004). In physiological conditions, Aβ40 is the main product. Still, dysregulation of γ-secretase complex catalytic subunits, such as APH1B, favors the generation of longer Aβ peptides, such as neurotoxic Aβ42 and Aβ43, in the brain (Acx et al., 2014; Park et al., 2021). On the other hand, the dysregulation of NCSTN was shown to influence the stability of γ-secretase-Aβ complexes. Thus, controlling the efficiency of the sequential proteolysis and thereby defining the length of Aβ products (Petit et al., 2019). This strongly supported the causative role of the high accumulation of toxic Aβ and dysregulation of the proteins involved in regulating the APP processing pathway in the presence of a mutation in the APP. In contrast, SIRT1 deacetylates substrates favored in the non-amyloidogenic pathway (Li et al., 2018). Molecular studies showed that SIRT1 activation prevents the accumulation of Aβ plaques and tau pathology through the NF-κB signaling pathway by upregulation of the *ADAM10* gene, induction of the Notch pathway, and inhibition of the mTOR pathway (Chen et al., 2005; Lee et al., 2014), indicating the protective role of SIRT1 in preventing the Aβ generation.

Between four isomers of tocopherol, we showed that only ATF and GTF reduced the level of Aβ42 in APP Swe-expressing cells. Ironically, no change was observed in the level of APP protein which is derived from the Aβ protein. Interestingly, the treatment with ATF and GTF activated the gene involved in the non-amyloidogenic pathway, such as *SIRT1*, and suppressed the genes involved in the amyloidogenic pathway, such as *BACE1*, *APH1B*, and *NCSTN*, especially in APP Swe/Ind-expressing cells, suggesting that the mechanism of action of these isomers is not necessarily directly on the APP protein expression. In addition, it also suggests that one of the possible mechanisms is *via* the regulation of the non-amyloidogenic or amyloidogenic APP processing pathway. ATF and GTF have been extensively studied as antioxidants in reducing ROS in oxidative stress-related diseases, including AD (Pahrudin Arrozi et al., 2020), and in scavenging reactive nitrogen species in the brains with AD (Williamson et al., 2002). This study is also in line with Gugliandolo et al. (2019), which conducted a transcriptional approach to investigate the effect of ATF treatment on the APP processing pathway. Their findings showed that ATF treatment increased the cell viability and upregulated the genes involved in the non-amyloidogenic processing of APP, such as *ADAM17* and *RTN4*, which is known to inhibit the β-secretase action. At the same time, it downregulated the gene involved in the amyloidogenic pathway, such as *PSEN2*, which is part of the γ-secretase in retinoic acid-differentiated SH-SY5Y neuroblastoma cells exposed to Aβ42. Different cell models with exogenous Aβ induction altered other genes with ATF treatment. However, the same effect was obtained, in which ATF shifted the gene expression profile toward the non-amyloidogenic pathway. Our results are also supported by previous works that characterize the non-antioxidant property of these tocopherol isomers as an anti-amyloid. For example, a study using AD transgenic (APPswe) model mice with alpha-tocopherol transfer protein knock-out showed increased Aβ deposits in the brain due to decreased Aβ clearance, which then ameliorated with ATF supplementation (Nishida et al., 2009). The findings from transcriptomic study showed strong alteration on genes expression in the hippocampus of albino rats fed with a vitamin E-deficient diet. Vitamin E which mainly comprises GTF directly or indirectly regulated an important number of genes including genes involved in the clearance of AB in order to ameliorate the burden of amyloid load thus, indicating the protective effect of GTF in the brain. On these genes to ameliorate the burden, indicating the protective effect of GTF in the brain against Aβ (Rota et al., 2005).

There are several limitations to this study. The *in vitro* cell models were not mimicking the exact pathological condition in the AD brain, and no *in vivo* study was conducted to support the findings. In addition, the anti-amyloidogenic property of ATF and GTF treatment was measured only in APP Swe-expressing cells as the level of Aβ42 was in between the APP WT and APP Swe/Ind-expressing cells. The effect of the treatment could be dependent on the amyloid load in the cells. Furthermore, we only compared two concentrations, and only for a 24 h treatment. A total dose- and time-dependent study would be more informative. The protein conformers of the Aβ42 was also not investigated to further explain the role of ATF and GTF treatment as an anti-amyloid. Moreover, this study only focuses on the possible mechanism exerted by ATF and GTF on the APP processing pathway at the gene level, not at the protein level. Confirming the expression at the protein level together with the enzymatic activity as the primary determining factor for the APP processing leading to Aβ production would be more conclusive.

## Conclusion

In conclusion, our results suggested that ATF and GTF could protect the cells against Aβ toxicity, possibly by activating and suppressing the genes expression involved in the regulation of APP non-amyloidogenic and amyloidogenic processing pathways, respectively, thus indicating that it may be useful in AD treatment.

## Data Availability Statement

The raw data supporting the conclusions of this article will be made available by the authors, without undue reservation.

## Author Contributions

SM designed the study and supervised the project. SNSS and APA performed the experiments and prepared the figures. SM, WZWN, and HAD provided the experimental technical assistance and commented on the results. APA, SNSS, NMM, ABAS, and SM wrote the manuscript. All authors contributed to the manuscript and approved the submitted version.

## Conflict of Interest

The authors declare that the research was conducted in the absence of any commercial or financial relationships that could be construed as a potential conflict of interest.

## Publisher’s Note

All claims expressed in this article are solely those of the authors and do not necessarily represent those of their affiliated organizations, or those of the publisher, the editors and the reviewers. Any product that may be evaluated in this article, or claim that may be made by its manufacturer, is not guaranteed or endorsed by the publisher.
